# Environmental protection tax and green innovation of heavily polluting enterprises: A quasi-natural experiment based on the implementation of China’s environmental protection tax law

**DOI:** 10.1371/journal.pone.0286253

**Published:** 2023-06-15

**Authors:** Juqiu Deng, Jiayu Yang, Zhenyu Liu, Qingyang Tan

**Affiliations:** School of Economics, Sichuan University, Chengdu, Sichuan, China; Usak University: Usak Universitesi, TURKEY

## Abstract

Environmental protection tax is an important tool for directing environmentally friendly growth in heavily polluting enterprises, but existing research has yet to provide consistent conclusions on whether and how environmental protection tax can promote green innovation in heavily polluting industries. The paper uses a double difference model based on data from Chinese listed companies in heavily polluting industries from 2012 to 2021 to empirically investigate whether environmental protection tax drives green innovation behavior of heavily polluting enterprises. The findings show that the environmental protection tax increases the degree of green innovation in heavily polluting enterprises, primarily through the anti-driving effect, in which an increase in environmental management expenses forces firms to increase their R&D investment, which improves the degree of green technical innovation. Furthermore, the environmental protection tax has a strong promotion effect on heavy polluters’ green innovation for state-owned enterprises and those in growing period or located in high marketization regions. However, this promotion effect is insignificant for non-state-owned enterprises and those in recession period, and environmental protection tax hinders green innovation of enterprises in mature period and those located in low marketization regions. Accordingly, it is suggested to improve preferential tax policies, increase investment in corporate green innovation and strengthen the supervision of environmental tax.

## 1. Introduction

Since the reform and opening up, China’s previous extensive economic development model has caused serious environmental pollution. To deal with the worsening pollution problem, China piloted the implementation of the pollutant charge fee system in 1979. However, there are some problems in the implementation of the system, such as low emission collection standards, insufficient rigidity of law enforcement and lack of standardization, which greatly reduce the effectiveness of pollution control. In order to overcome the disadvantages of the system and more effectively implement the concept of green development, the Chinese government introduced an environmental protection tax on January 1, 2018, which is the first separate tax law for environmental protection in China. As a more coercive and executive instrument of environmental regulation, the environmental protection tax plays a positive role in forcing enterprises to transform and upgrade, promoting the adjustment of economic structure and development mode to the intensive type and realizing green development0. It cannot be ignored that the environmental protection tax may affect the green technology innovation of enterprises by affecting the investment cost of enterprises. The improvement of green total factor productivity is the fundamental way to solve the problem of the dual pressure of environmental protection and economic growth in China, and the improvement of green productivity mainly depends on the green technological innovation of enterprises [1]. So how does the introduction of the environmental protection tax affect the green innovation of enterprises? Is the effect positive or negative? Is there heterogeneity in this effect? An in-depth study of the impact of environmental protection tax on enterprises’ green innovation is not only conducive to exploring the "win-win" model of economic development and environmental protection, but also of great significance in promoting enterprises to achieve green transformation.

Environmental protection tax is a typical tool for environmental regulation, while at present there are differences in the research conclusions on the role of environmental regulations on enterprises’ green innovation. The traditional view of the neoclassical school of economics holds that the imposition of environmental protection tax will increase the costs of enterprises and reduce the profits of them, which will hinder the innovation activities of enterprises. Using the Chinese Carbon Emissions Trading Pilot Policy as a quasi-natural experiment, C et al. [2] found that this environmental regulation policy reduces enterprises’ economic performance, thereby reducing the R&D investment, making overall enterprise technology innovation weaker. The empirical results of Stucki et al. [3] suggest that environmental laws and regulations will reduce the innovation tendency of enterprises if taxes and regulations cannot generate additional demand. However, the Porter Hypothesis [4] holds that the appropriate environmental regulation will stimulate the green technological innovation of enterprises, because the economic results of the green technological innovation of enterprises can offset the cost of environmental tax and compensate for the input of green innovation, thus increasing the profits of enterprises. Acemoglu et al. [5] believe that proper environmental regulation tools can achieve enterprises’ green technology innovation, supporting the Porter Hypothesis. Peng et al. [6] found that environmental regulations enhances the enthusiasm of enterprises for green innovation and thus promotes green innovation behavior. The research of Zhang et al. [7] and Hu et al. [8] also show a positive relationship between environmental regulation and green innovation. But beyond that, some scholars believe that the impact of environmental regulation on corporate innovation is uncertain or not obvious [9, 10]. In this paper, we argue that the reason for the dispute is that the influence of environmental taxes and fees on enterprises’ green innovation is the result of the combination of the crowding out effect and the anti-driving effect, while the extent of them is uncertain.

To answer the above questions and test the net effect of environmental protection tax on enterprises’ green innovation, this paper constructs a three-sector model including consumers, producers and government based on Yu Pang’s research [11] and theoretically analyzes the relationship between environmental protection tax and enterprises’ green innovation. On this basis, this paper takes the Chinese A-share listed companies in heavily polluting industries from 2012 to 2021 as a sample, and uses the double difference model to test the net effect of environmental protection tax on enterprises’ green innovation. The results show that: (1) the environmental protection tax significantly improves the level of enterprises’ green innovation, and the conclusion is still significant after parallel trend test, PSM, replacement of explained variables and so on. (2) The influence of environmental protection tax on green innovation of enterprises is heterogeneous, which plays a positive role in state-owned enterprises, mature enterprises and enterprises located in areas with high level of marketization, but it plays an inhibiting role in growing enterprises and enterprises in areas with low level of marketization. In non-stated-owned enterprises and enterprises in recession period, the impact of environmental protection tax on the level of enterprises’ green innovation is not significant.

The following are this paper’s marginal contributions: First, it enriches the theoretical framework of environmental protection tax. By building a theoretical model, we derive the conditions under which the environmental protection tax encourages green innovation in enterprises. Most of the existing research examines the influence of environmental protection taxes on corporate green innovation from an empirical perspective, but few do so from the perspective of theoretical modeling. Second, it broadens research into the economic effects of environmental protection taxes. The existing findings are mostly concerned with environmental governance and corporate environmental investment, with little attention paid to the influence of environmental levies on green innovation at the micro-level. Third, it optimizes the method for measuring the effect of environmental protection tax. The paper develops a double difference model based on the quasi-natural experiment of "Environmental Protection Fee to Tax" in China, which effectively alleviates the endogeneity problem of reverse causation in OLS empirical study. Fourth, it improves the accuracy of the study of the effect of an environmental protection tax. In this study, we focus on heavily polluting enterprises, the core target group of the environmental tax, and further explore the impact of the environmental tax on heavy polluters with different ownership, life cycle status and located in different marketization regions, providing empirical evidence for the government to clarify the impact of the environmental tax as a regulatory instrument and provide more targeted environmental protection policies.

## 2. Literature review

### 2.1 Study on the effects of environmental taxes

In 1920, Pigou first proposed the notion of environmental taxes and constructed a theoretical framework [12].Environmental taxes internalize the external costs of enterprises [13] to correct the negative externalities of environmental contamination. Scholars’ study on the effect of environmental taxes and fees as an environmental control instrument mostly focuses on the "double dividend" effect [14], i.e., environmental dividend and economic dividend, however, the conclusions of existing theoretical and empirical studies are inconclusive as to whether the "double dividend" can be realized.

Some scholars have verified the presence of the "double dividend" effect, in which environmental taxes and fees can encourage emission reduction and GDP growth [15], and are more efficient than distortionary taxes and subsidies in improving social welfare [16–18]. Some scholars support the existence of the first dividend, i.e., the environmental dividend. Taking the United States [19, 20], Europe [21], China [22], and Japan [16] as examples, they demonstrate that the implementation of carbon taxes and etc. help effectively cut emissions and improve the ecological environment. The existence of the second dividend, i.e., the economic dividend, is still debated in extant studies, with some scholars finding that the implementation of environmental taxes has no significant effect on the country’s economic efficiency [23] and even has a detrimental impact on the economy and the residents welfare [24]. Bovenberg and Mooij suggest that the existence of the second dividend depends on the size of the revenue recycling effect and the tax interaction effect [25].

### 2.2 Study on green innovation

Green innovation mainly refers to green technology innovation, and related study originated in the 1990s. E. Brown and D. Wield first proposed the notion of green technology in 1994, which refers to any technologies connected to environmental improvement, including technologies, processes and products that avoid or decrease pollution and utilize resources [26]. Thereafter, academics have carried out a wealth of research on green technology innovation, and its definition has been progressively perfected [27, 28]. The World Intellectual Property Organization (WIPO) introduced the International Patent Classification Green Inventory on September 16, 2010, and it is currently the most precise and comprehensive definition of green innovation. It assigns green innovation technologies specific green patent numbers and covers nearly all environment-related pollutant treatment technologies, resource-saving technologies, and climate change mitigation technologies.

Most scholars have investigated green innovation in two stages, innovation input stage and innovation output stage, and have chosen different indicators to reflect the input and output respectively. For example, the environmental R&D budget is used to measure innovation input, the pollution level is used to measure innovation output [29], R&D input is used to represent innovation input, and the number of patent applications is used to represent innovation output [30]. Also, based on the system assessment level, some researchers argue that the design of a green innovation performance evaluation index should cover three aspects including green product innovation performance, economic performance and environmental performance [31]. In terms of the factors driving green innovation, the extant literature focuses on enterprise competition [32], government subsidies [3, 33], finance [34], and environmental regulation [4].

### 2.3 Study on environmental regulation and enterprise green innovation

The impact of environmental regulation on green innovation has always been a frontier topic, with many experts conducting detailed research, but no agreement has been achieved. According to the Porter Hypothesis, appropriate environmental regulation (particularly market-based tools) can compel enterprises to engage more in green innovation, resulting in more innovative activities. In the long term, the efficiency gains from green technologies and tax incentives might offset the negative costs of environmental regulation, resulting in an "innovation compensation effect" [4].

Based on studies in Germany [35], Finland [36] and other continental European countries [37], some scholars have supported the Porter Hypothesis that environmental regulation have a positive impact on firms’ green innovation. Government regulation, as represented by environmental taxes and fees, motivates enterprises to engage in green innovation [38], and the stronger the environmental policy adjustment and regulation, the greater the incentive for firms to engage in green innovation activities [39]. Some other scholars have discovered that environmental regulation have a negative impact on firms’ green innovation. On the one hand, environmental regulation increase enterprises’ environmental costs [40], equipment costs [41], and tax burden [42], reducing profits and production incentives; on the other hand, it causes the diversion of funds for green innovation [43], thus preventing enterprises from carrying out green technological innovation activities. And some scholars argue that the impact of environmental regulation on green innovation is complex rather than a simple linear relationship, such as Lanoie et al. [44], who argue that environmental regulation and green innovation have a U-shaped relationship.

### 2.4 Literature description and evaluation

The rich research results mentioned above have laid a solid foundation for this paper, but there is still room for further research in the following aspects: First, China began implementing the environmental protection tax law in 2018.There are few studies on the micro impact of environmental taxes on Chinese enterprises, and the analysis methods are primarily empirical studies, with few literature using a dual combination of developing theoretical models and conducting empirical studies to investigate the impact of environmental protection taxes. Second, most empirical studies on environmental taxes and green innovation use the number of patents to measure green innovation, failing to reflect the green attributes of innovation, while this paper intends to improve it by using data of green patents. Third, current studies have primarily used quantitative indicators such as emission fees and environmental taxes to measure environmental protection taxes, which have subjective and endogenous concerns. For instance, more environmental taxes and fees are paid with larger production scales, while they may also produce more innovation as a result of their larger size. Therefore, imposing emission fees is not always a cause of promoting green innovation among enterprises. The implementation of the environmental protection tax in China is an exogenous event shock, providing a quasi-natural experiment for this paper to investigate the policy impacts of the environmental protection tax.

## 3. Theoretical analysis and research hypothesis

According to the Porter Hypothesis, in the case of environmental taxation, if a firm’s fixed production cost stays the same, it will consider whether to engage in green innovation activities to lower pollution emissions and boost profits.

Based on Porter Hypothesis, this paper creates a three-sector model that comprises consumers, producers and government by following Yu Pang [11]. Consumers consume agricultural and industrial goods while being exposed to environmental pollution, assuming consumers’ utility function is:

$$\begin{array}{c} U=u(C_{A})+u(C_{M})+v(E)=C_{A}+\alpha C_{M}-\frac{\beta }{2}C_{M}{}^{2}-\gamma E\\ \left(\alpha ,\beta ,\gamma >0\right) \end{array}$$ (1)

*C*_*A*_ and *C*_*M*_ stand for consumer consumption of industrial and agricultural commodities respectively, while *E* stands for overall pollution emissions. The utility function *u*(*C*_*M*_) is monotonically increasing and concave to the origin, whereas the utility function v(E) is monotonically declining, which is:

$$\frac{\partial u}{\partial C_{M}}>0,\frac{\partial^{2}u}{\partial C_{M}{}^{2}}<0,\frac{\partial v}{\partial E}<0$$ (2)

Assuming that consumers perform labor inelastically and earn wages of *W*; that the government levies a tax of *T* on producers of industrial goods and uses it to subsidize consumers; that the price of agricultural products is unitized to 1 and the price of industrial goods relative to agricultural products is *p*, the budget constraint line for consumers is:

$$W+T=C_{A}+pC_{M}$$ (3)

Assuming no information asymmetry and equal production and sales in the industrial products market, the reverse demand function for industrial goods under consumer utility maximization can be constructed from Eqs (3-1) and (3-2) (since the consumer utility function is linear, the transfer payment *T* does not affect the demand for industrial goods):

$$p=p(C_{M})=\alpha -\beta C_{M}$$ (4)

The manufacturing sector consists of *N*(*N*>1) homogeneous production companies. Manufacturing companies will generate pollutants as by-products while producing, and each unit of output will generate *θ* = *θ*(*λ*) units of pollutants. *λ* is the green production technology used by the company, and while *λ* = 0, *θ* = *θ*′, *θ* function monotonically decreases and 0<*θ* = *θ*(*λ*)≤*θ*′.That is, with the company’s conventional production, each unit of output will generate *θ*′ units of pollutants. If the company uses green production technology, the pollutants generated per unit of output is less than *θ*′, and the more advanced the technology, the smaller the amount of pollutants generated per unit of output.

The environmental tax is levied based on the enterprise’s pollutant emissions, and the tax amount per unit of emissions is *t*. Let *f*(*q*) represents the enterprise’s production cost and let *q* represents the enterprise’s normal production output, i.e., the highest output at the lowest cost that the enterprise can accomplish with the existing scale and equipment under normal production conditions. Since the usage of green production technology may affect the enterprise’s production efficiency, the production efficiency is set to *A* = *A*(*λ*). *A*(*λ*).≥1 and is monotonically increasing. While *λ* =0, meaning that the enterprise does not employ green production technology, *A* = 1. The enterprise’s actual production output equals to *q*×*A*(*λ*).

When the production enterprise does not employ green production technology, the enterprise’s profit before tax is:

$$\begin{array}{l} \pi_{1}=p_{1}Q_{1}-f(Q_{1})-t\theta_{1}Q_{1}\\ \;=(\alpha -\beta q)q-t\theta^{\prime }q-f(q) \end{array}$$ (5)

If the production enterprise chooses to develop the green innovation technology on their own, let the R&D cost of the green production technology be *C* = *C*(*λ*) and $\frac{\partial c}{\partial \lambda }>0$. The enterprise’s profit before tax is:

$$\pi_{2}=p_{2}Q_{2}-f(Q_{2})-C(\lambda )-t\theta_{2}Q_{2}$$ (6)

Bringing the yields *Q*_2_ = *A*(*λ*)*q*, *p*_2_ = *α*−*βA*(*λ*)*q*,*θ*_2_ = *θ*(*λ*) into Eq (3-6), get:

$$\pi_{2}=[\alpha -\beta A(\lambda )q]A(\lambda )q-C(\lambda )-tq\theta (\lambda )A(\lambda )-f[A(\lambda )q]$$ (7)

When the enterprise’s pre-tax profits from utilizing green production technologies exceed those from not using them (*π*_2_>*π*_1_), the enterprise autonomously performs R&D on green innovation technologies, leading to:

$$t\left[\theta^{\prime }-\theta (\lambda )A(\lambda )\right]>[A(\lambda )-1]\{\beta q[A(\lambda )+1]-\alpha \}+\frac{C(\lambda )+f[A(\lambda )q]-f(q)}{q}$$ (8)

According to the above equation, when the following conditions satisfied,

$$\theta^{\prime }-\theta (\lambda )A(\lambda )>0$$ (9)

$$t>\frac{\left[A(\lambda )-1]\{\beta q[A(\lambda )+1]-\alpha \}q+C(\lambda )+f[A(\lambda )q]-f(q\right)}{q[\theta^{\prime }-\theta (\lambda )A(\lambda )]}$$ (10)

The adoption of green production technologies boosts corporate profits primarily through the decrease of pollutant emissions in production enterprises.


$$\mathrm{When}\;\theta^{\prime }-\theta (\lambda )A(\lambda )<0$$ (11)

$$t<\frac{\left[A(\lambda )-1]\{\beta q[A(\lambda )+1]-\alpha \}q+C(\lambda )+f[A(\lambda )q]-f(q\right)}{q[\theta^{\prime }-\theta (\lambda )A(\lambda )]},$$ (12)

The primary way in which green production technology increases the profit of the enterprise is by enhancing production efficiency. In the short term, it is considered that the normal production output *q*, the amount of pollution per unit of output under conventional production *θ*′, the cost function *f*(*q*) and the cost of green innovation *C*(*λ*) of production enterprises are unchanged, thus whether production enterprises engage in green innovation activities is connected to the burden of environmental tax, the production efficiency of green production technology and the amount of pollution per unit of output after adopting green production technology. The findings listed above demonstrate that environmental protection tax is a significant factor in enterprises’ green innovation. On this basis, this paper argues that environmental protection tax has "crowding-out effect" and "innovation compensation effect" enterprises’ green innovation.

On the one hand, the "cost hypothesis" indicates that, assuming a static game, once the government strengthens environmental regulation, the cost of environmental management for industrial businesses will rise inevitably, resulting in a "crowding-out effect" on R&D investments and therefore a negative impact on innovation [18]. The most immediate effect of the environmental tax is that it raises enterprises’ production costs and reduces their cash flow. This will lower the R&D investments in green innovation and inhibit green innovation, i.e., the environmental tax will have a "crowding-out effect" on green innovation.

**Fig 1:**
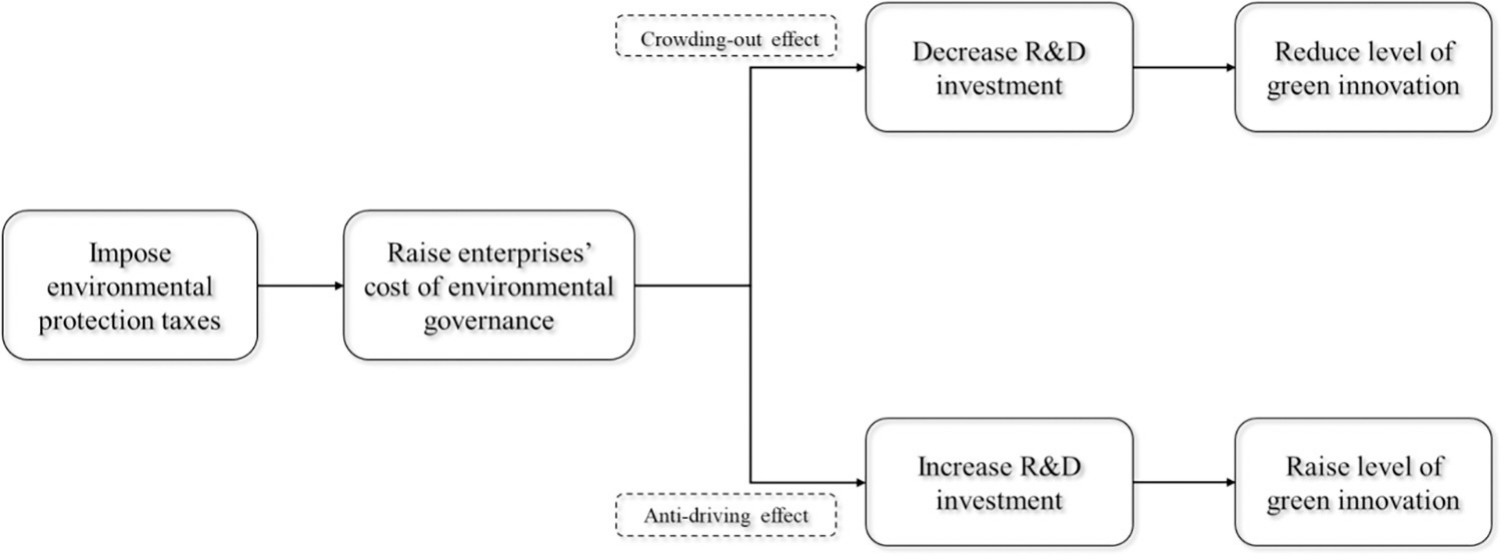
The influencing mechanism of environmental protection taxes on green innovation.

According to Porter’s "innovation compensation" theory, this article thinks that environmental protection taxes have an "anti-driving effect" on green innovation. The cost of pollution control in heavily polluting industries will climb in the short term, lowering enterprises’ profit margins. To lower the cost of pollution control, businesses will aggressively change their production and management strategies, expand R&D and innovation activities and improve the technological content and environmental quality of their products. In the long run, these innovations will offset the cost of environmental protection by improving productivity and reducing environmental taxes. According to Porter’s "first-mover advantage" hypothesis, in the context of economic development shifting to a resource-saving and environmentally friendly economy, some medium and heavy polluting firms will be the first to carry out green innovation activities in order to save costs and improve benefits, thus gaining a competitive advantage [18]. Therefore, this paper proposes that environmental taxes may drive firms to boost green innovation investment and therefore stimulate green innovation, i.e., environmental taxes have an "anti-driving impact". Fig 1 depicts the mechanism of environmental protection influencing green innovation.

## 4. Research design

### 4.1 Sample selection and data sources

In 2018, China’s Environmental Protection Tax Law went into effect. To effectively identify policy effects and account for data availability, this paper selects A-share listed companies in heavily polluting industries from 2012 to 2021 as samples. We limit the research object to the heavily polluting industries because they are more sensitive to environmental protection taxes. According to the classification standard of the *Classified Management Directory of Listed Companies in the Environmental Protection Verification Industries* issued by the Ministry of Environmental Protection of China in 2008, heavily polluting industries include the following 14 industries: thermal power, iron and steel, cement, electrolytic aluminum, coal, metallurgy, building materials, mining, chemical, petrochemical, pharmaceutical, light industry, textile and leather. Therefore, the samples of heavily polluting enterprises selected in this paper are from listed companies in the above 14 industries, excluding ST and *ST enterprises and those with missing data. We then collect samples and their financial statistics from the CSMAR database and WIND database. Data on green patents granted are collected from the WIND database based on WIPO’s International Patent Classification Green Inventory. Data of corporate environmental protection governance in the mechanism analysis are manually compiled based on the construction-in-progress schedules of listed companies’ annual reports. Furthermore, Stata15 is used to process the empirical results.

### 4.2 Explanation of variables

#### 4.2.1 Explained variable

Total number of green inventions obtained (Hdzl). Green innovation encompasses both green innovation input and output. In the domestic empirical research, green innovation input is frequently measured by indicators such as environmental protection R&D capital and personnel investment, related tangible and intangible asset investment, while green innovation output are frequently measured by pollution level, energy consumption per unit product, and environment-related patent data. However, because of the difficulties in distinguishing between indicators of green innovation input and enterprise innovation input, this paper only conducts an empirical study from the perspective of green innovation output, and uses the number of green patents granted to measure enterprises’ green innovation level.

#### 4.2.2 Explaining variable

Environmental Protection Tax (DID = Fgs×Time). During the implementation of environmental protection tax, some provinces used the original emission fee levy standard as the standard of environmental protection tax, while 12 provinces, including Hebei, Henan, Jiangsu, Shandong, Hunan, Sichuan, Chongqing, Guizhou, Hainan, Guangxi, Shanxi, and Beijing, increased this standard. From this, Fgs is used as the grouping variable: if the standard of environmental protection tax in the province where the enterprise is located is raised, Fgs is 1 (Treatment group), otherwise is 0 (Control group). Time is set as a time variable, and as the environmental protection tax goes into effect on January 1, 2018, Time is 0 prior to 2018, and 1 from 2018 to 2021.

#### 4.2.3 Control variables

To decrease estimate bias caused by omitted variables, this paper draws on the existing literature and selects a series of variables that may affect corporate green innovation including corporate size, gearing ratio, profitability, cash flow ratio, years of corporate listing, shareholding ratio of the first largest shareholder, equity checks and balances, average monthly excess turnover ratio, shareholding ratio of institutional investors, shareholding ratio of management, management fees ratio, capital appropriation by major shareholders and whether the Big Four. Table 1 provides detailed definitions.

**Table 1:** The definition of variables.

Variable Symbol	Variable Name	Variable Description
Hdzl	Total number of green inventions obtained	Total number of green inventions obtained
DID	Interaction items	Grouping dummy variable × Time dummy variable
Fgs	Grouping dummy variable	Fgs = 1, if a province’s standard of environmental protection tax is increased, and Fgs = 0 otherwise
Time	Time dummy variable	Before 2018 (pre-implementation), Time = 0; in the year of implementation and thereafter, Time = 1
Size	corporate size	Natural logarithm of corporate’s total assets
Lev	Gearing ratio	Total liabilities/total assets
Roe	Profitability	Profit before tax/total assets
Cashflow	cash flow ratio	Net cash flows from operating activities/total assets
FirmAge	years of corporate listing	Natural logarithm of (current year—year of company establishment + 1)
Top1	shareholding ratio of the first largest shareholder	Number of shares held by the first largest shareholder/total number of shares
Balance	equity checks and balances	The sum of the shareholdings of the second to fifth largest shareholders / the shareholding of the first largest shareholder
Dturn	average monthly excess turnover ratio	Average monthly stock turnover this year-Average monthly stock turnover last year
INST	shareholding ratio of institutional investors	Total number of shares held by institutional investors/outstanding share capital
Mahsare	shareholding ratio of management	Number of shares held by management/total number of shares
Mfee	management fees ratio	Management fees/operating income
Occupy	capital appropriation by major shareholders	Other receivables/total assets
Big4	whether the big four	Big4 = 1 if the company is audited by PWC, KPMG or Ernst & Young, and Big4 = 0 otherwise

## 5. Empirical analysis

### 5.1 Descriptive statistics

Table 2 displays the descriptive statistics for the main variables. The total number of green inventions obtained (hdzl) has a maximum value of 484, a minimum value of 0, a mean value of 2.048 and a standard deviation of 10.292, showing a large fluctuation generally, which means that there are significant differences between different heavily polluting enterprises’ levels of green innovation. Among the control variables, the statistics of enterprise size (size) and management expense ratio (mfee) differ comparatively.

**Table 2:** Descriptive statistics of the main variables.

Variable	Obs	Mean	Std. Dev.	Min	Max
Hdzl	8526	2.048	10.292	0	484
DID	8526	0.069	0.254	0	1
Fgs	8526	0.42	0.494	0	1
Time	8526	0.167	0.373	0	1
Size	8526	22.075	1.353	17.537	28.636
Lev	8526	0.474	0.202	0.007	1.262
Roe	8526	0.039	0.545	-29.144	21.348
Cashflow	8526	0.058	0.076	-0.762	0.6
Firmage	8526	2.676	0.395	1.099	3.689
Top1	8526	0.375	0.158	0.003	0.9
Balance	8526	0.594	0.573	0	3.989
Dturn	8526	-0.069	0.484	-6.886	3.987
Inst	8526	0.331	0.251	0	1.87
Mshare	8526	0.071	0.161	0	0.98
Mfee	8526	0.094	2.749	-170.249	185.429
Occupy	8526	0.021	0.072	0	3.959
Big4	8526	0.065	0.246	0	1

### 5.2 Analysis of regression results

Table 3 shows the regression results of the baseline model0. Column (1) contains no control variables, and the coefficient of DID is 10.081 and significant at the 10% level, demonstrating that the environmental protection tax encourages green technology innovation among heavily polluting enterprises0. Column (2) includes time and industry fixed effects to eliminate the effect of the target and implementation time differences, and the findings reveal that the estimated coefficient of DID grows and is considerably positive at the 5% level. Column (3) continues to include control variables, and the coefficient value of DID remains considerably positive at the 5% level and the R^2^ rises. These results and improved model fit support the validity of the H1b hypothesis from the preceding section.

**Table 3:** Basic regression results.

Variable	Hdzl		
	(1)	(2)	(3)
DID	1.081*	1.185**	1.202**
	(0.598)	(0.589)	(0.567)
Fgs	0.422*	0.332	0.103
	(0.244)	(0.241)	(0.232)
Time	4.052***	7.538***	4.679***
	(0.386)	(0.776)	(0.886)
Size			1.988***
			(0.113)
Lev			-3.139***
			(0.626)
Roe			-0.304
			(0.198)
Cashflow			-2.557*
			(1.457)
Firmage			-0.56
			(0.365)
Top1			2.262**
			(1.086)
Balance			0.667**
			(0.269)
Dturn			0.18
			(0.259)
Inst			0.774
			(0.605)
Mshare			0.357
			(0.82)
Mfee			-0.003
			(0.038)
Occupy			5.465***
			(1.518)
Big4			3.541***
			(0.468)
_Cons	1.119***	-0.228	-41.34***
	(.158)	(7.091)	(7.238)
Year Fixed Effects	No	Yes	Yes
Industry Fixed Effects	No	Yes	Yes
Observations	8526	8526	8526
R-Squared	0.028	0.062	0.134

Note

Standard errors are in parentheses.

***,** and * indicate statistical significance at the levels of 1%,5% and 10% respectively. The same applies the following tables.

### 5.3 Robustness test

#### 5.3.1 Parallel trend test

To use the double difference method, the parallel trend assumption must be met, which states that the treatment and control groups must maintain the same trend prior to the policy shock and that there is a significant difference between them after the policy shock. In this study, we constructed the following parallel trend test model based on Eq (4-1).


$$Hdzl_{it}=\overset{4}{\underset{k=-5}{\Sigma }}\alpha_{i}DID_{it}+\beta_{j}{\mathrm{Control}}_{it}+\gamma_{i}+\mu_{t}+\varepsilon_{it}$$ (13)

**Fig 2:**
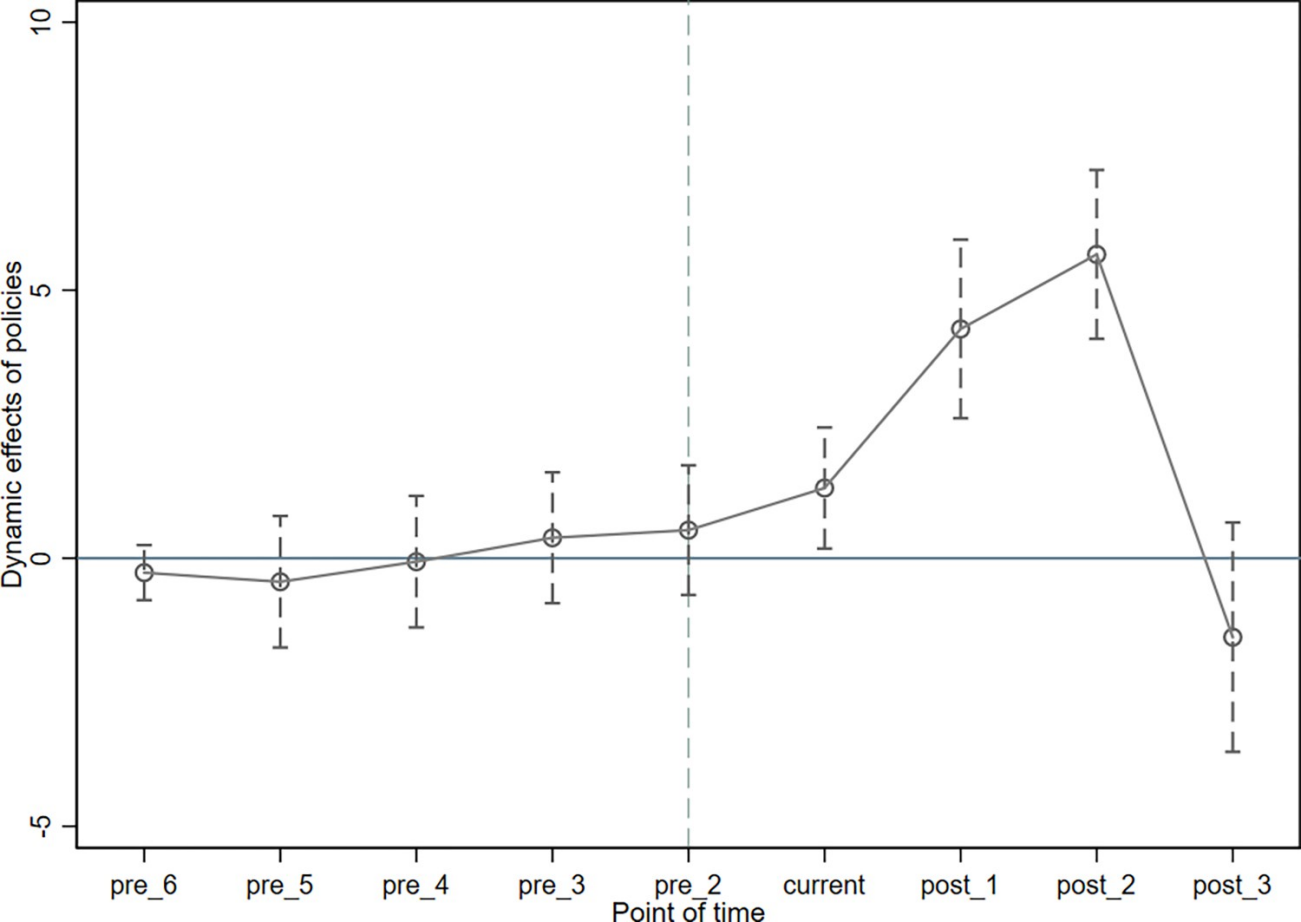
Parallel trend test.

In Eq (2), the subscript k specifies the number of periods that deviate from the base period. The analysis’ findings are shown in Fig 2, and it is clear that before the policy was implemented, the estimated coefficients of the policy effects were not significant and fluctuated around 0. This suggests that the variation trend in the treatment and control groups prior to the increase in the environmental protection tax rate standard remained consistent. While following the implementation of the policy, the total number of green inventions obtained by heavily polluting enterprises in the treatment group region grew significantly compared to the control group, and the samples passed the parallel trend test, allowing the use of the double difference approach.

#### 5.3.2 Replacement variables and PSM-DID

The subsequent robustness tests were carried out in this research to prevent bias caused by inability to account for the impact of possibly unobservable variables: Use the total number of green invention applications (sqzl) as a new indicator of the level of green innovation among heavily polluting enterprises. Use the Logit model and K-nearest neighbor matching approach to propensity score matching and perform regression on the matched 5377 samples.

Table 4 displays the results of the regression. The results of the double difference method’s regression using the total number of green inventions applications as the explanatory variable are shown in Columns (1) and (2), and the estimated coefficients of the interaction term DID before and after adding the control variables are positive and significant at the 10% level. The results of the double difference method’s regression using propensity score matching are shown in Column (3), and the coefficient of DID is significant at the 10% level. The coefficient estimated are consistent with the previous paper, confirming the robustness of the finding that the environmental protection tax increases the level of green innovation among heavily polluting enterprises.

**Table 4:** Robustness check.

Variable	Sqzl		
	DID	DID	Psm+DID
	(1)	(2)	(3)
DID	1.18*	1.152*	1.368*
	(0.673)	(0.645)	(0.718)
Fgs	0.374	0.108	
	(0.262)	(0.252)	
Time	11.106***	7.081***	
	(0.898)	(1.01)	
Control Variables	Control	Control	Control
Year Fixed Effects	Yes	Yes	Yes
Industry Fixed Effects	Yes	Yes	Yes
_Cons		-47.214***	-77.912***
		(7.868)	(14.254)
Observations		8372	5377
R-Squared		0.158	0.205

#### 5.4 Mechanism inspection

The above-mentioned baseline regression results and robustness tests demonstrate that environmental protection taxes aid in the improvement of green innovation in heavily polluting enterprises. It is necessary to investigate if the environmental protection tax has a "crowding-out effect" or an "anti-driving effect" on green innovation. We then make regression analysis using environmental protection expenses and R&D investment of heavily polluting enterprises as the explanatory variable respectively. Environmental protection expenses(lnzfy), containing 26 specific items such as environmental protection facilities and equipment, environmental protection technology improvement, wastewater facilities, waste gas facilities, dust removal facilities and dust rising facilities is defined as the natural logarithm of (the amount of enterprises’ environmental protection investment × 100) / shareholders’ equity balance. R&D investment (lnyfzc) is defined as the natural logarithm of enterprises’ R&D investment. The regression results are shown in Columns (1)-(2) of Table 5. The above findings indicate that the environmental protection tax has a n "anti-driving effect" on heavily polluting enterprises’ green innovation, i.e., the legal pressure to comply with the environmental protection tax raises their environmental management cost and forces them to increase their R&D investment, which improves their green innovation level.

**Table 5:** Mechanism inspection results.

	(1)	(2)
	Lnzfy	Lnyfzc
DID	0.345**	0.002***
	(0.164)	(0.001)
Control Variables	Control	Control
Year Fixed Effects	Yes	Yes
Industry Fixed Effects	Yes	Yes
_Cons	-5.492***	-0.104***
	(1.798)	(0.016)
Observations	2325	6452
R-Squared	0.424	0.193

## 6. Further discussion

### 6.1 Grouping by enterprise ownership

Enterprises’ investment choices are influenced by the nature of property rights. To investigate the impact of the environmental protection tax levy on heavily polluting enterprises with different ownership, this study splits the samples into two categories for regression: state-owned enterprises(SOEs) and non-state-owned enterprises(non-SOEs), with the results presented in columns (1)-(2) of Table 6.

**Table 6:** Heterogeneity analysis: Corporate ownership.

Variable	Hdzl	
	(1)	(2)
DID	4.44***	-0.864
	(0.878)	(0.765)
Fgs	0.532	-0.648*
	(0.325)	(0.36)
Time	5.962***	4.167***
	(1.278)	(1.397)
Control Variables	Control	Control
Year Fixed Effects	Yes	Yes
Industry Fixed Effects	Yes	Yes
_Cons	-48.689***	-26.744***
	(7.635)	(4.455)
Observations	4337	3838
R-Squared	0.234	0.06

As shown in Table 6, SOEs have a DID coefficient that is highly positive at the 1% level, showing that the environmental protection tax greatly encourages these businesses to implement green innovation. The reasons might be: On the one hand, green technological innovation has high public value attributes, and SOEs, as the major form of public ownership economy, bear high-level social responsibility. The government’s commitment to combat pollution in places where the environmental tax rate has been raised is strong and SOEs face more urgent pressure for green transformation, so they are more motivated to increase invest in green innovation. On the other hand, SOEs have the advantage of special resources, more reliable sources of capital and abundant human capital reserves, so they are more capable of bearing the long-term investment cost of technological innovation and consequently more willing to continuously supply research and development for green innovation.

The regression results for non-SOEs are insignificant, demonstrating that imposing the environmental protection tax has little effect on heavily polluting non-SOEs’ willingness to engage in green innovation. This could be because heavily polluting non-SOEs face greater marginal costs of pollution control, and the economic output from green innovation can hardly balance the firms’ environmental control expenses, making it difficult for the environmental protection tax to provide them with a significant incentive to innovate. Furthermore, as heavily polluting non-SOEs have inadequate financing capacity, limited financial and technological strength and unstable production and operation situation, it is hard to achieve green transformation in the short term. Therefore, the policy shock of imposing the environmental protection tax has essentially no influence on heavily polluting non-SOEs.

### 6.2 Grouping by enterprise life circle

An enterprise’s life cycle is the dynamic trajectory of its development and expansion, with distinct management modes and organizational structure forms at various times. These differences have an impact on how motivated and resource-intensive enterprises are to carry out green innovation in response to the environmental protection tax. In this study, we divide the samples into three groups: the growing period group, the mature period group and the recession period group The findings are shown in columns (1)-(3) of Table 7. We can see that as enterprises develop and expand, the impact of environmental taxes on the degree of green innovation in heavily polluting enterprises presents inhibition during the growing period, significant promotion during the mature period and no significant influence during the recession period.

**Table 7:** Heterogeneity analysis: Enterprise life cycle.

Variable	Hdzl		
	(1)	(2)	(3)
DID	-2.518**	4.188***	0.905
	(1.037)	(0.983)	(0.9)
Fgs	0.101	-0.095	0.22
	(0.428)	(0.396)	(0.372)
Time	6.779***	3.086**	5.225***
	(1.633)	(1.489)	(1.453)
Control Variables	Control	Control	Control
Year Fixed Effects	Yes	Yes	Yes
Industry Fixed Effects	Yes	Yes	Yes
_Cons	-38.427***	-43.141***	-40.234***
	(4.938)	(4.399)	(8.439)
Observations	1848	2705	3952
R-Squared	0.145	0.199	0.12

The cause is that green innovation normally needs enterprises to commit a lot of money and time. In the growing period, enterprises have no nature profit model and low net cash flow, and tends to allocate capital to projects that are short, quick and have steady return. Facing the increase of pollution control costs caused by environmental taxes, they are hampered by a lack of resources and R&D experience and therefore more likely to purchase environmental protection equipment to reduce pollution instead of green innovation. In the mature period, enterprises have advantages in profitability, reputation, size and R&D capital, and the issue of endogenous and exogenous financial restrictions is partially mitigated. When faced with severe enforcement of environmental taxes, their willingness to cope with tax pressure through novel pollution-fighting technology while maintaining their dominant position and market share with higher green patent holdings will be stronger. In the recession period, due to limited cash flow, lagging technological innovation efficiency and capacity for applying research results to production, rigid and conservative organizational structures and the deviation from consumer preferences, enterprises are more likely to choose to use their excess cash flow to cover operating losses or pay back loans and dividends than to invest in green technological advancements.

### 6.3 Grouping by level of marketization

As a market-based regulatory device, the influence of environmental taxes on heavily polluting enterprises’ green innovation is inextricably linked to the level of marketization in the region in which they are located. Based on the marketization index, we split the samples into two groups: higher level of marketization group and lower level of marketization group. The findings are shown in columns (1)–(2) of Table 8. It is discovered that the implementation of the environmental protection tax encourages green innovation activities greatly of heavily polluting enterprises in areas with a high level of marketization, but inhibits green innovation activities to those in areas with a low level of marketization.

**Table 8:** Heterogeneity analysis: Marketization index.

Variable	Hdzl	
	(1)	(2)
DID	2.287**	-1.56***
	(0.956)	(0.494)
Fgs	0.225	-0.077
	(0.446)	(0.178)
Time	2.856**	5.542***
	(1.367)	(0.647)
Control Variables	Control	Control
Year Fixed Effects	Yes	Yes
Industry Fixed Effects	Yes	Yes
_Cons	-65.677***	-23.838***
	(13.056)	(5.751)
Observations	4154	4372
R-Squared	0.194	0.132

The reason might be: In more marketized regions, the market resource allocation impact dominates and the price mechanism responds more smoothly. Enterprises not only pursue environmental legitimacy of stakeholders such as the government, creditors and shareholders, but also prioritize the allocation of idle resources in response to the cost stickiness effect of environmental taxes and actively alter their business strategies for greener development. At the same time, the market incentive, constraint and trading mechanisms in more marketized regions function more smoothly, and the intellectual property protection system is also more perfect, which facilitates the optimal allocation of innovation resources and provides a good environmental for the realization of the innovation compensation effect, so that heavily polluting enterprises are more ready to make green innovation in the face of policy shock of environment taxes. In less marketized regions, the anti-driving effect of environmental protection taxes on the green transformation of businesses is less pronounced because the government resource allocation effect predominates and the market mechanism cannot operate in an ideal environment.

## 7. Conclusion

As a market-incentivized environmental regulation strategy, environmental protection taxes encourage heavily polluting enterprises to fulfill their environmental duties and accelerate their green transformation. Using a sample of A-share listed firms in heavily polluting industries from 2012 to 2021, this research gives an empirical examination of the influence of environmental protection tax on the level of green innovation in heavily polluting enterprises. The following are the key findings:

1. The implementation of environmental protection taxes promotes heavily polluting enterprises to advance green technologies.
2. The impact of environmental protection taxes on heavily polluting firms’ green innovation is exerted through the "anti-driving effect," in which an increase in environmental management costs encourages enterprises to boost R&D investment, hence improving their green innovation level.
3. Further research reveals that the promotion effect of environmental protection taxes on green innovation of heavily polluting enterprises exists significantly in state-owned enterprises, mature enterprises, and enterprises in regions with high level of marketization, but is insignificant for non-state-owned enterprises and enterprises in recession period and inhibits enterprises in growing period and those in regions with low level of marketization.

Based on the above conclusions, the policy significance of this research mainly lies in the following:

First of all, preferential tax policies should be further improved. The heavily polluting enterprises carrying out green innovation activities will generally face relatively great financial pressure. The government should make full use of proper preferential tax policies to increase the benefits of green innovation in heavily polluting enterprises while collecting environmental taxes, especially to reduce the tax burden of enterprises that develop low carbon, energy saving and new energy products.

Secondly, it is necessary to strengthen the financial support for green innovation. Capital is one of the crucial factors influencing the level of green innovation in enterprises. It is suggested that special management of environmental tax should be carried out, and the revenue of environmental tax should be exclusively used for environmental pollution control and financial support for enterprises to develop environmental friendly technologies and projects.

Finally, the government should strengthen the supervision of environmental tax and evaluate its implementation effect. The government should attach importance to the collection and management of environmental tax, implement the policy of environmental tax reduction and exemption in earnest, and constantly improve the environmental tax system, in order to maximize its incentive effect on green innovation of heavily polluting enterprises.

## Supporting information

S1 Dataset (DTA)
