# Correction: Environmental protection tax and green innovation of heavily polluting enterprises: A quasi-natural experiment based on the implementation of China’s environmental protection tax law

**DOI:** 10.1371/journal.pone.0315030

**Published:** 2024-12-02

**Authors:** Juqiu Deng, Jiayu Yang, Zhenyu Liu, Qingyang Tan

The first two authors, Juqiu Deng and Jiayu Yang, should be noted as shared first co-authors and contributing equally to this work.
